# Internal hernia strangulated on appendicular tourniquet: a case report of an exceptional anomaly of the appendix revealed by a rare hernia

**DOI:** 10.1186/s40792-019-0671-0

**Published:** 2019-07-15

**Authors:** Ismaïl Lawani, Cocou Yélinhan Patrice Houndje, Yacoubou Imorou Souaïbou, Dansou Gaspard Gbéssi, Setondji Gilles Roger Attolou, Freddy H. R. Gnangnon, Kouègnigan Sylvain Komadan, Houénoukpo Koco, Francis Moïse Dossou, Jean-Léon Olory-Togbé

**Affiliations:** 1 Department of Surgery and Surgical Specialties, Faculty of Health Sciences, Campus Universitaire Champs de Foire, PO Box: 01BP118, Cotonou, Republic of Benin; 2 Faculty of Medicine, University of Parakou, PO Box: BP 123, Parakou, Republic of Benin

**Keywords:** Internal hernia, Appendicular tourniquet, Bowel obstruction

## Abstract

**Background:**

Internal hernias and the appendicular tourniquet are two rare pathologies.

**Case presentation:**

We report here a case of a 68-year-old obese patient, who has acute small bowel obstruction due to strangulated internal hernia through an appendicular tourniquet. This appendicular tourniquet results from the adhesion between the tip of the appendix and its body. This obstruction was complicated by plugged perforation. Resection of the small bowel segment was performed, in addition to appendectomy, lavage, and drainage. The postoperative recovery was uneventful.

**Conclusion:**

Acute bowel obstruction secondary to strangulation of an internal hernia through an appendicular tourniquet is exceptional.

## Introduction

Some forms of appendicitis can cause bowel obstruction [1, 2]. Among these forms, some authors have described the appendicular tourniquet [3, 4]. It is an ileocecal appendix that forms a loop in which the small bowel can penetrate and perform an internal hernia. Internal hernias correspond to a gut exit through a natural or abnormal intra-abdominal orifice [5]. They are often revealed by an occlusive syndrome of which they constitute a rare etiology [6]. We report here the case of a patient operated for bowel obstruction complicated of small bowel perforation, whose etiology found intraoperatively, and an internal hernia on an appendicular tourniquet. The interest of this case lies in the exceptional and novel character of this association of two rare pathologies.

## Case description

**Fig. 1:**
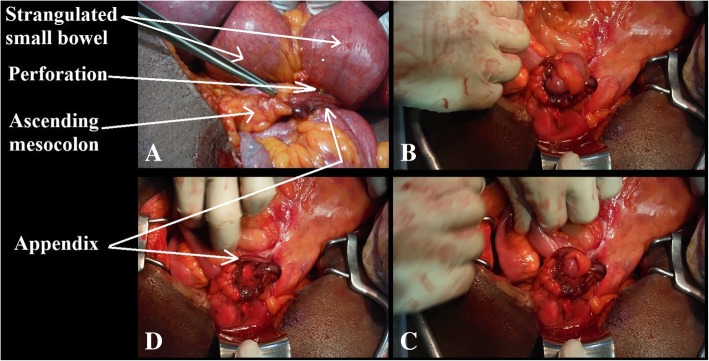
**a** Appendicular tourniquet enclosing a small bowel; the forceps show the zone of adhesion between the ileocecal appendix and the expansion of the ascending mesocolon. **b**, **c** Reduction of the internal hernia. **d** Appendicular loop after reduction

**Fig. 2:**
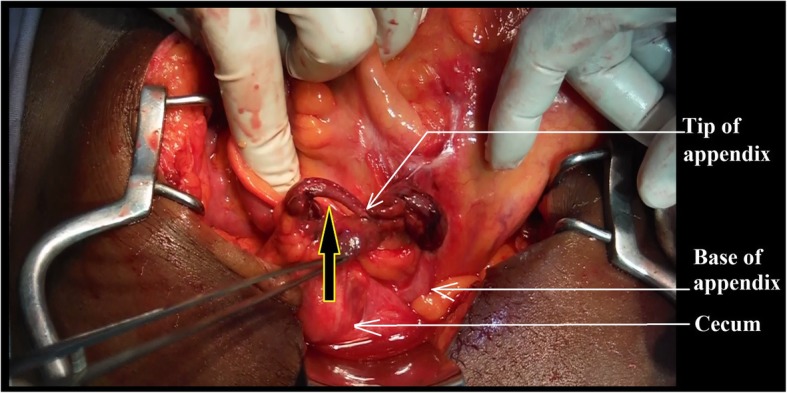
The orifice formed by the appendicular tourniquet designated by the black arrow

It was a 68-year-old obese patient with a body mass index of 35 kg/m^2^, who had emergency consultation for an occlusive syndrome that had been evolving for three days prior to admission. He was complaining of abdominal pain, emesis, and cessation of passage of gas and stool. The pain had begun in the epigastrium. On examination, there was a normal temperature of 37.3 °C and a distended and flexible abdomen. The umbilical and inguinal hernia orifices were free and the rectal bulb empty. A gastroscopy performed the day before the hospitalization had concluded to an extrinsic mass compressing the gastric body associated with nodular congestive bulbopathy. An occlusion on a probable tumor of the transverse colon is evoked and a laparoscopy is first decided to locate the tumor and decide of a colectomy in one or two stages. Anesthesia was general with orotracheal intubation. We used one 10-mm optical port into the umbilical and one five-mm trocar into the right hypochondrium. At the laparoscopy, there is no colonic tumor but a significant distention of the small bowel leading to a perilous exploration. We decide to convert. At laparotomy, we discover an internal hernia of the strangulated small bowel in an orifice newly formed by the ileocecal appendix, with a plugged perforation in the throttling groove (Fig. 1a). The middle third of the appendix body had fused with the ascending mesocolon (Fig. 1a). When herniated loops are reduced (Fig. 1b, c), the tip of the appendix is found to have returned to merge with its body, forming a loop in which the small bowel had herniated (Fig. 1 d) and (Fig. 2). The emptying of the small intestine was successively achieved by the opening of the perforation located about 60 cm from the ileocecal valve, the segmental resection of the small bowel carrying the perforation, the immediate restoration of continuity by ileo-ileal end-to-terminal anastomosis by two hemi-sites of vicryl 3/0 reinforced by separate points, appendectomy, and washing and drainage of the right iliac fossa. The postoperative treatment consisted on ceftriaxone 2 g/day for five days, metronidazole 1.5 g/day, paracetamol 3 g/day for 10 days, omeprazole 20 mg/day, enoxaparin 0.4 ml/day, parenteral nutrition with oliclinomel® at the rate of 2 l/24 h for five days, and continuous digestive aspiration by nasogastric tube. Ablation of the nasogastric tube was done on the fifth day after resumption of transit and the patient resumed oral feeding. The patient returned home on the eighth postoperative day. Histological examination of the operative specimen revealed acute phlegmonous appendicitis. The patient was seen at three months and then at one year without complications.

## Discussion

The first three cases of small bowel obstruction due to appendicitis were described in 1901 by Hotchkiss [7]. Since then, several authors have published other cases, which have made it possible to classify the occlusions of the small bowel in relation to an appendicitis in several groups. Thus, according to Soo and Tsegha [8] and Makama et al. [1], we distinguish the paralytic ileus, mesenteric ischemia, mechanical occlusions without strangulation, and finally the mechanical occlusion by strangulation, and the first case was described by Naumov [9] in 1963. The term appendicular tourniquet means the loop resulting from the adhesion of the tip of the appendix to its base in the manner of a tourniquet. This is a rare pathological situation. In 2016, Chowdary [4] identified only 16 published cases. Adhesion of the appendicular tip is thought to be related to the inflammatory process and peritoneal reaction in response to appendicitis [8]. The diagnosis is almost always intraoperative. Indeed, in most cases, the occlusive syndrome dominates the clinical situation, thus masking the signs of appendicitis. This was also the case of our patient. The occlusion in this case is by internal hernia of the small intestine in the appendicular loop and constitutes the circumstance of discovery. The initial hypothesis in our patient was tumor occlusion of the transverse colon. The importance of distension explains the impression of extrinsic compression that was visualized at the gastroscopy and misplaced our diagnosis even more. The abdominal computed tomography would have been a better help. Indeed, it is the most appropriate examination to explore the occlusive syndrome [10] because it specifies the mechanism, the seat, and very often the cause. It would probably have allowed preoperative diagnosis and would have made it possible to avoid exploratory laparoscopy, carried out here for diagnostic purposes, in the hypothesis of the colonic tumor. However, computed tomography can be unavailable, especially into resource-limited hospitals in low-income countries such as ours. Waiting for that imaging can then lead to complications that are more difficult to treat like perforation followed by peritonitis. In our case, there was no peritonitis because the perforation has been blocked by the appendicular knot. According to Sebastian-Valverde et al. [11], a laparoscopic approach in the management of small bowel obstruction is feasible, effective, and safe. It has been clinically proven to be an advantage over an open approach [12]. It is associated with better postoperative outcomes, lower morbidity, an earlier onset of oral intake, and a shorter length of hospital stay [11]. However, it requires a specific skill set, it may not be appropriate in all patients [13], and patient selection is the strongest key factor for having success [11]. Conversion to median laparotomy is becoming increasingly rare in countries with high technical equipment and the list of conversion patterns is becoming limited [14]. The best exploration of the peritoneal cavity is no longer a motive for conversion. However, the fragility of the distended loops and the risk of iatrogenic perforation were the main reason that led to the conversion in our case. The incision in the right iliac fossa is not suitable to treat the appendicular node. As in our case, all authors used medial laparotomy as providing a better day to treat occlusion and its cause. Treatment always consists on an appendectomy associated or not with intestinal resection—suture as a function of the viability of the small intestine [1–4, 7–9].

## Conclusion

The appendicular tourniquet is an extremely rare complication of appendicitis. It is most often revealed by an internal hernia and its diagnosis is almost always intraoperative.

## Data Availability

Data sharing is not applicable to this article as no datasets were generated or analyzed during the current study.
